# Research on the intervention effect of Five-Element Music combined with Eight-Section Brocade on depression among medical students in higher vocational colleges

**DOI:** 10.3389/fpsyg.2024.1439713

**Published:** 2024-09-30

**Authors:** Xiyong Yao, Lin Xiong, Yiwen Ouyang, Hui Wang, Lili Zhu

**Affiliations:** ^1^College of Traditional Chinese Medicine, Chongqing Medical and Pharmaceutical College, Chongqing, China; ^2^College of Music, Mahasarakham University, MahaSarakham, Thailand; ^3^College of Traditional Chinese Medicine, Chongqing Medical University, Chongqing, China; ^4^College of the Arts, Guangxi University, Nanning, China

**Keywords:** Five-Element Music, Eight-Section Brocade, medical students in higher vocational colleges, depression intervention, Music and Baduanjin for depression

## Abstract

**Background:**

Depression is the main risk factor leading to suicidal ideation among college students. This study focused on observing and assessing how the combination of Five-Element Music and Eight-Section Brocade affected depression levels among medical students attending higher vocational colleges.

**Methods:**

From a total of 1,030 medical students studying in higher vocational colleges, we selected 160 students who showed depressive symptoms and met the specific criteria for the study. We used the SDS scale to identify these students and made them the participants of our experiment. Participants were randomly divided into four groups: the music intervention group (listening to traditional Chinese Five-Element Music for 15 min daily), the Eight-Section Brocade intervention group (practicing the Eight-Section Brocade exercises once daily, approximately 15 min), the combined intervention group (first practicing the Eight-Section Brocade exercises once, then listening to music for 15 min), and the control group (no intervention). Each group consisted of 40 participants. The three intervention groups (excluding the control group) underwent continuous intervention for 4 weeks. The SDS, SAS, and PSQI scales were used for evaluation before and after the intervention.

**Results:**

Except for the control group, the SDS, SAS, and PSQI scores of the other three groups were lower after the intervention than before the intervention (*p* < 0.01). After the treatment, the scores on SDS, SAS, and PSQI tests did not vary much between the students who listened to music and those who practiced Eight-Section Brocade (the difference was not statistically significant, *p* > 0.05). However, the students who did both music and Eight-Section Brocade showed significantly lower scores than those who did only one activity (both *p* < 0.01).

**Conclusion:**

Five-Element Music and Eight-Section Brocade can improve depression, anxiety, and sleep status among medical students in higher vocational colleges. The combined intervention of the two is more effective than a single method, and it is worth promoting and applying in higher vocational colleges.

**Clinical Trial Registration:**

https://www.chictr.org.cn/showproj.html?proj=210705.

## Introduction

Depression, characterized by negative emotions, is the most prevalent psychological issue among university students and a major risk factor for suicidal ideation (He and Yang, 2015). The incidence of depression among medical students in higher vocational colleges is higher compared to undergraduate and graduate students (Xiong and Wan, 2019), and it is on the rise, necessitating urgent intervention. Currently, interventions for depression and other psychological issues among university students often focus on psychological regulation or physical exercise. In clinical settings, Traditional Chinese Medicine (TCM) interventions have been widely applied and proven effective in treating depression related to various conditions. Numerous successful cases demonstrate that TCM offers invaluable methods for the prevention and management of mental health issues. Combining Five-Element Music with the Eight-Section Brocade, a traditional Chinese health exercise, has shown good results in helping people with depression caused by different illnesses. But, we do not see many studies using this combo to help university students, especially medical students in vocational colleges, with their depression. Music therapy is straightforward and can be easily done, and many medical students already practice the Eight-Section Brocade. So, it’s very practical and easy to use these two methods to help medical students in vocational colleges with their depression. This study investigates the use of Five-Element Music and the Eight-Section Brocade, both separately and combined, to alleviate depression among medical students in vocational colleges. It observes and assesses the effects of these interventions, aiming to offer fresh perspectives and techniques for combating depression among university students. The following report outlines the findings.

## Subjects and methods

### Study subjects

#### Source of study subjects

A total of 1,030 sophomore students from the Colleges of Chinese Medicine, Clinical Medicine, Pharmacy, and Nursing at Chongqing Medical and Pharmaceutical College were selected. The students were evaluated in their intact classes using the Self-Rating Depression Scale (SDS) for screening purposes. Among them, 160 students with depressive symptoms (standard score ≥ 53) were identified as study subjects and randomly assigned into four groups using a random number table method. Each group consisted of 40 students: the Chinese Medicine Five-Element Music Intervention Group (hereinafter referred to as the Music Intervention Group), the Chinese Medicine Health-Preserving Exercise Eight-Section Brocade Intervention Group (hereinafter referred to as the Eight-Section Brocade Intervention Group), the Combined Intervention Group that combined both Five-Element Music and Eight-Section Brocade, and a Control Group. The Music Intervention Group included nine male and 31 female students with a mean age of 19.23 ± 0.92 years. There were 28 cases of mild depression, nine cases of moderate depression, and three cases of severe depression, with a mean depression score of 60.98 ± 7.01. The Eight-Section Brocade Intervention Group had eight male and 32 female students with a mean age of 19.40 ± 0.59 years. Among them, there were 29 cases of mild depression, eight cases of moderate depression, and three cases of severe depression, with a mean depression score of 60.40 ± 6.29. The Combined Intervention Group consisted of nine male and 31 female students with a mean age of 19.47 years. It included 28 cases of mild depression, nine cases of moderate depression, and three cases of severe depression, with a mean depression score of 60.73 ± 7.14. The Control Group had nine male and 31 female students with a mean age of 19.33 ± 0.66 years. Among them, there were 29 cases of mild depression, eight cases of moderate depression, and three cases of severe depression, with a mean depression score of 60.25 ± 5.69. There were no statistically significant differences in gender, age, grade, depression severity, and mean depression scores among the four groups (*p* > 0.05), indicating comparability.

#### Inclusion criteria

(1) Self-Rating Depression Scale (SDS) score ≥ 53; (2) Willing to participate in randomly assigned intervention groups; (3) Not participating in other intervention training besides the assigned group; (4) Adherence to intervention principles and completion of intervention training sessions as prescribed; (5) Informed consent obtained from intervention subjects, with signed informed consent forms.

#### Exclusion criteria

(1) SDS score <53; (2) Unwillingness to undergo intervention training; (3) Failure to complete training sessions as prescribed for any reason; (4) Inability to participate in post-intervention assessments for any reason; (5) Refusal to sign informed consent forms.

#### Dropout criteria

(1) Accumulated failure to adhere to intervention requirements on three occasions during the intervention process; (2) Voluntary withdrawal from the intervention midway due to non-cooperation; (3) Premature termination for any other reasons.

### Intervention methods

1. Five-Element Music Intervention Group: The music selected is “China’s Traditional Wuxing (Five Elements) Therapeutic music (Zhengdiao), (Medium Tune)” performed by The National Central Musical College (one of China’s top orchestras.), published by CHINESE MEDICAL MULTIMEDIA PRESS, composed by Shi Feng. The music library includes tunes of Jue, Zhi, Gong, Shang, and Yu, totaling five tunes. The selection of appropriate Five-Element Music is based on syndrome differentiation by 2–3 chief TCM physicians or above. For Liver-Qi stagnation syndrome, tunes in the Yu mode are used; for Liver-Qi transforming into Fire syndrome, tunes in both Yu and Yu modes are used; for Phlegm-Qi stagnation syndrome, tunes in the Yu and Gong modes are used; for Spleen and Heart deficiency syndrome, tunes in the Zhi and Gong modes are used; for Heart-Nourishment deficiency syndrome, tunes in the Zhi mode are used; for Heart-Kidney Yin deficiency syndrome, tunes in the Zhi and Yu modes are used; for Spleen-Kidney Yang deficiency syndrome, tunes in the Gong and Yu modes are used. Once daily, 15 min each time, continuously for 4 weeks.
2. Eight-Section Brocade Intervention Group: The theoretical and routine practices of the Eight-Section Brocade exercises are taught by specialized instructors from the School of TCM. Students learn and proficiently master the theoretical principles and routines of the exercises. The full set of exercises is practiced once daily, approximately 15 min each time, continuously for 4 weeks. Participants with more than three absences (including three times) are considered ineffective interventions and are not statistically processed.
3. Combined Intervention Group of Five-Element Music and Eight-Section Brocade: After practicing the Eight-Section Brocade exercises, participants listen to Five-Element Music. The content of each intervention method is the same as that of the Eight-Section Brocade group and the Five-Element Music group.
4. Control Group: No intervention is conducted.

### Research instruments

The Self-Rating Depression Scale (SDS), Self-Rating Anxiety Scale (SAS), and Pittsburgh Sleep Quality Index (PSQI) were used as research tools for the experimental subjects. They were also used to assess intervention effects before and after intervention. Each scale includes instructions for completion and basic information (such as name, gender, age, major, class, telephone number). The Self-Rating Depression Scale (SDS) was used to screen 160 students with depressive symptoms from a total of 1,030 students for inclusion in the study. After random allocation into four groups, the Self-Rating Anxiety Scale (SAS) and Pittsburgh Sleep Quality Index (PSQI) were used to assess their anxiety and sleep quality. At the end of the experimental intervention, all four groups of students were assessed using the SDS, SAS, and PSQI. The intervention effects were evaluated by comparing data before and after the intervention.

#### Self-rating depression scale (SDS)

Developed by William W.K. Zung in 1965, the SDS comprises 20 items, including two items for psychic affective symptoms, eight items for somatic symptoms, two items for psychomotor symptoms, and eight items for depressive psychological symptoms. Each item is rated on a 7-point scale. The scale is easy to use and provides a direct reflection of the subjective experience of depressed individuals, making it suitable for adults with depressive symptoms. Based on Chinese normative data, the cut-off score for the SDS standard score is 53. Scores ranging from 53 to 62 indicate mild depression, 63–72 indicate moderate depression, and scores of 73 or above indicate severe depression. Current research on the SDS has shown that the correlation coefficients for all 20 items are greater than 0.7. The test–retest reliability for each item ranges from 0.730 to 1.000, and the Cronbach’s *α* coefficient is between 0.782 and 0.784.

#### Self-rating anxiety scale (SAS)

Developed by Professor William W.K. Zung, a Chinese American, in 1971, the SAS consists of 20 items. The main rating criterion is the frequency of symptoms defined by each item, which is divided into four levels. There are 15 positively rated items and five negatively rated items. The total score of the 20 items is multiplied by a coefficient of 1.25, and the integer part of the result is considered the standard score. A score of 50 on the SAS is used as the cut-off value for the presence of anxiety. Scores ranging from 50 to 59 indicate mild anxiety, 60–69 indicate moderate anxiety, and scores above 70 indicate severe anxiety. In this study, the reliability analysis of the Self-rating Anxiety Scale revealed a standardized Cronbach’s *α* coefficient of 0.81.

#### Pittsburgh sleep quality index (PSQI)

The PSQI was developed by Dr. Buysse and colleagues from the Department of Psychiatry at the University of Pittsburgh in 1989. The PSQI consists of seven dimensions including sleep quality, sleep duration, sleep latency, sleep efficiency, sleep disturbances, use of sleep medications, and daytime dysfunction, totaling nine items. Each item is scored on a 4-point scale from 0 to 3, with higher scores indicating poorer sleep quality. The total score is the sum of scores from each dimension. The Cronbach’s *α* coefficient for this scale is 0.81.

### Quality control

Before the project initiation, uniform training is provided to the project team members and relevant supervising teachers. The content, experimental plan, objectives, and significance of the entire research project are clearly defined, and strict supervision is maintained over the specific intervention measures. Consistency in intervention methods is ensured within the same intervention group. Each group is assigned 1–2 supervising teachers to guide and supervise the students in completing the corresponding intervention measures daily. Additionally, 3–4 student leaders are designated for each group to organize the students in completing the corresponding intervention measures and to record the completion status through videos, photographs, etc. The self-rating scales are filled out in a centralized manner. Before filling out the scales, the supervising teachers uniformly explain the precautions to ensure the accuracy of the responses. After completion, the supervising teachers check the completeness of the questionnaires and collect them on the spot.

### Statistical methods

The data obtained were analyzed using SPSS 23.0 software. For quantitative data that met the criteria of normal distribution, results are presented as ( $\bar{x}$ ± S); for data that did not meet the criteria of normal distribution, results are presented as M ± Q. Based on the characteristics of the data, comparisons between groups were conducted using analysis of variance (ANOVA) and the Kruskal-Wallis H test. Comparisons within groups were conducted using paired t-tests and the Wilcoxon signed-rank test. The significance level for all tests was set at *α* = 0.05 (two-tailed).

### Research process


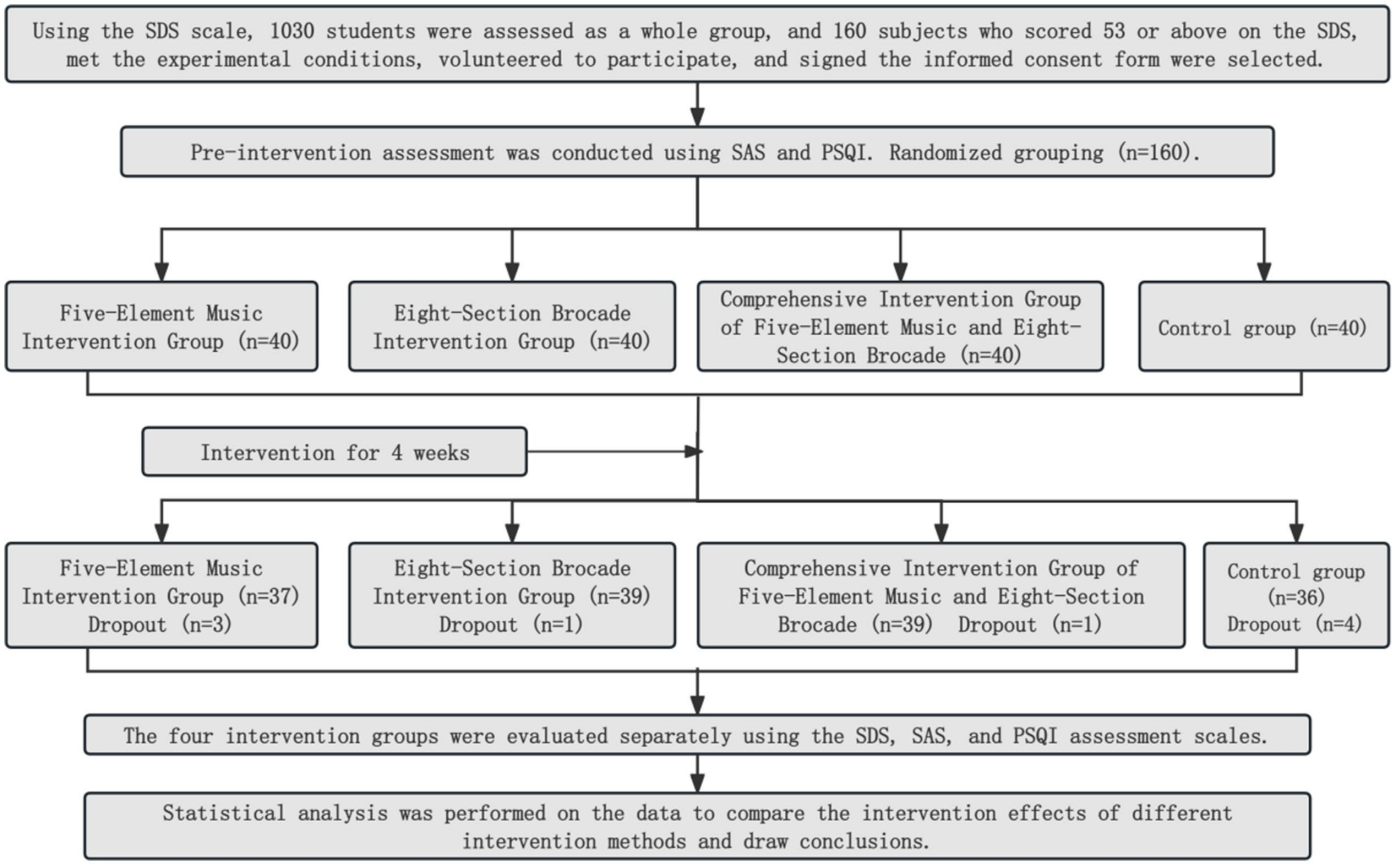


## Results

### Completion of the study

In the control group, there were four dropouts and 36 completions; in the Five-Element Music intervention group, there were three dropouts and 37 completions; in the Eight-Section Brocade intervention group, there was one dropout and 39 completions; in the Combined Intervention group, there was one dropout and 39 completions.

### Comparison of SDS scores before and after intervention in the four groups

**Figure 1:**
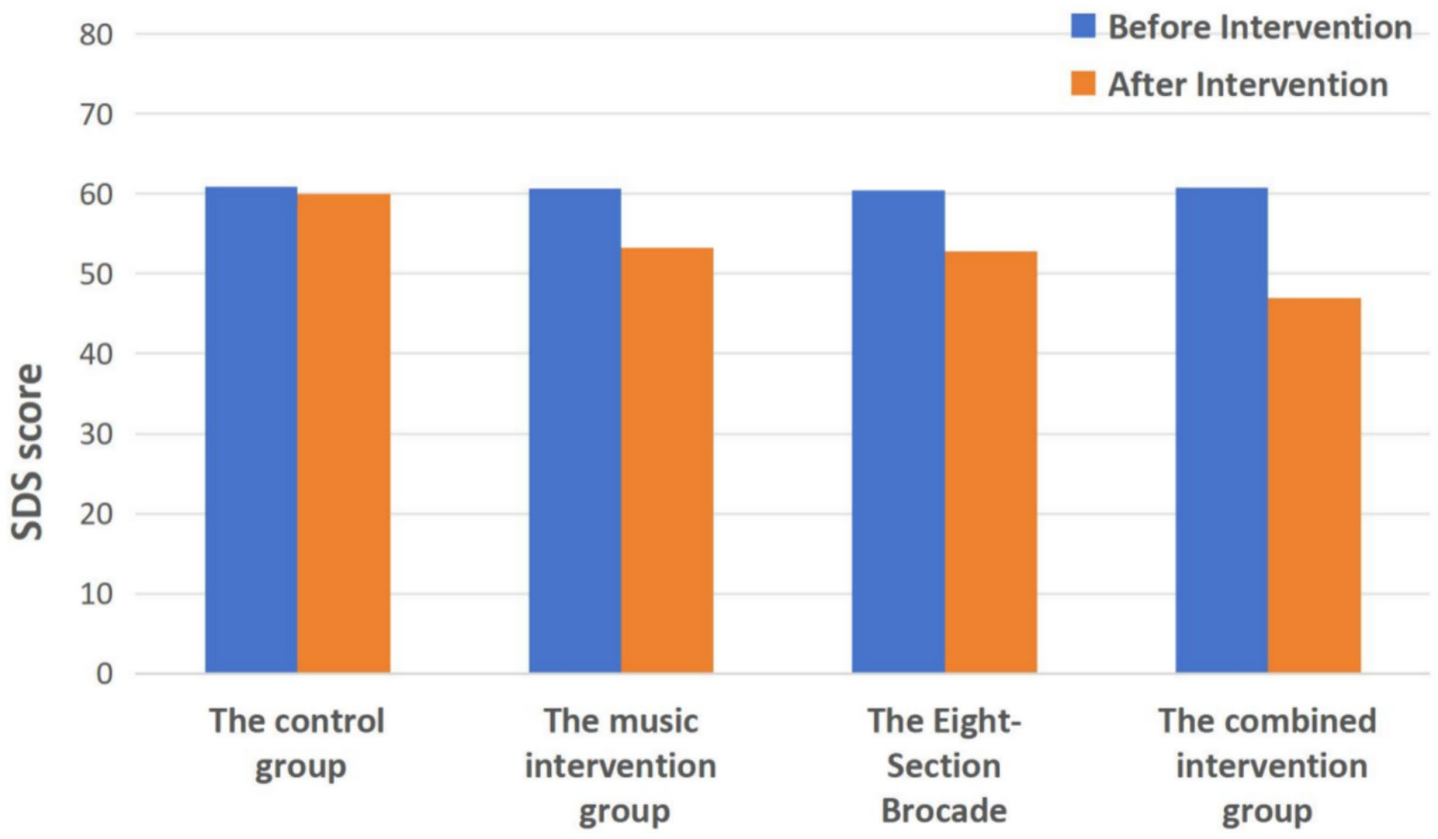
Comparison of SDS scores before and after intervention in the four groups.

As shown in Table 1 and Figure 1, before the intervention, there was no statistically significant difference in SDS scores among the four groups (*p* > 0.05). After the intervention, compared with before the intervention, the SDS scores in the Music group, Eight-Section Brocade group, and Combined group all significantly decreased (*p* < 0.01). After the intervention, the SDS score in the Combined group was lower than that in the Music group and Eight-Section Brocade group (both *p* < 0.05), while there was no statistically significant difference between the Music group and Eight-Section Brocade group (*p* > 0.05).

**Table 1:** Comparison of SDS scores before and after intervention in the four groups (Score, $\bar{x}$ ± SD).

Group	Number of cases	Before intervention	After intervention	t/z	p
The control group	36	60.861 ± 5.586	60.028 ± 5.438	1.631	0.112
The music intervention group	37	60.568 ± 7.061	53.222 ± 8.191△△**	5.566	0.001
The Eight-Section Brocade	39	60.385 ± 6.438	52.846 ± 7.506△△**	−4.667	0.001
The combined intervention group	39	60.795 ± 7.219	46.923 ± 6.979△△**#	11.863	0
*F*/*X*2	–	0.868	49.129	–	–
p	–	0.833	0	–	–

△△ Compared to before intervention within this group, *p* < 0.01; ** compared to after intervention in the control group, *p* < 0.01; #compared to after intervention in the Music intervention group and Eight-Section Brocade intervention group, *p* < 0.05.

### Comparison of SAS scores before and after intervention in the four groups

**Figure 2:**
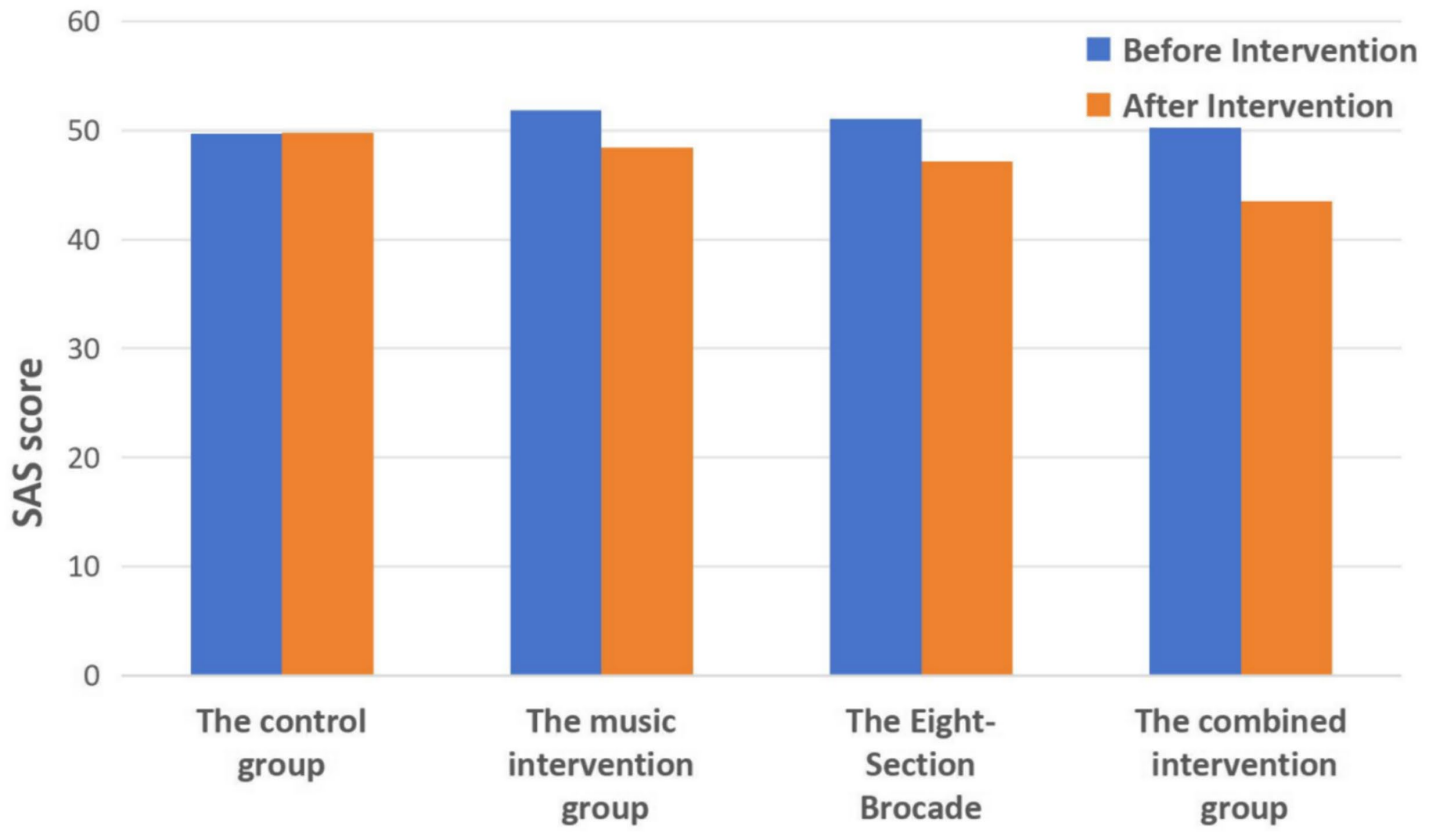
Comparison of SAS scores before and after intervention in the four groups.

As shown in Table 2 and Figure 2, before the intervention, there was no statistically significant difference in SAS scores among the four groups (*p* > 0.05). After the intervention, compared to before the intervention within each group, the SAS scores in the Music group, Eight-Section Brocade group, and Combined group all significantly decreased (*p* < 0.01). After the intervention, the SAS score in the Combined group was lower than that in the control group (*p* < 0.01), but there was no statistically significant difference between the Music group and Eight-Section Brocade group compared to the control group (*p* > 0.05). Additionally, there was no statistically significant difference in SAS scores between the Combined group and the Music group or Eight-Section Brocade group (*p* > 0.05).

**Table 2:** Comparison of SAS scores before and after intervention in the four groups (Score, $\bar{x}$ ± SD).

Group	Number of cases	Before intervention	After intervention	t/z	p
The control group	36	49.694 ± 10.116	49.806 ± 9.058	−0.094	0.925
The music intervention group	37	51.865 ± 8.709	48.417 ± 7.915△△	3.871	0
The Eight-Section Brocade	39	51.077 ± 9.001	47.179 ± 9.397△△	3.547	0.001
The combined intervention group	39	50.256 ± 6.528	43.487 ± 6.947△△**	5.672	0
*F*/*X*2	–	0.720	9.287	–	–
p	–	0.868	0.026	–	–

△△ Compared to before intervention within this group, *p* < 0.01; ** compared to after intervention in the control group, *p* < 0.01.

### Comparison of PSQI scores before and after intervention in the four groups

**Figure 3:**
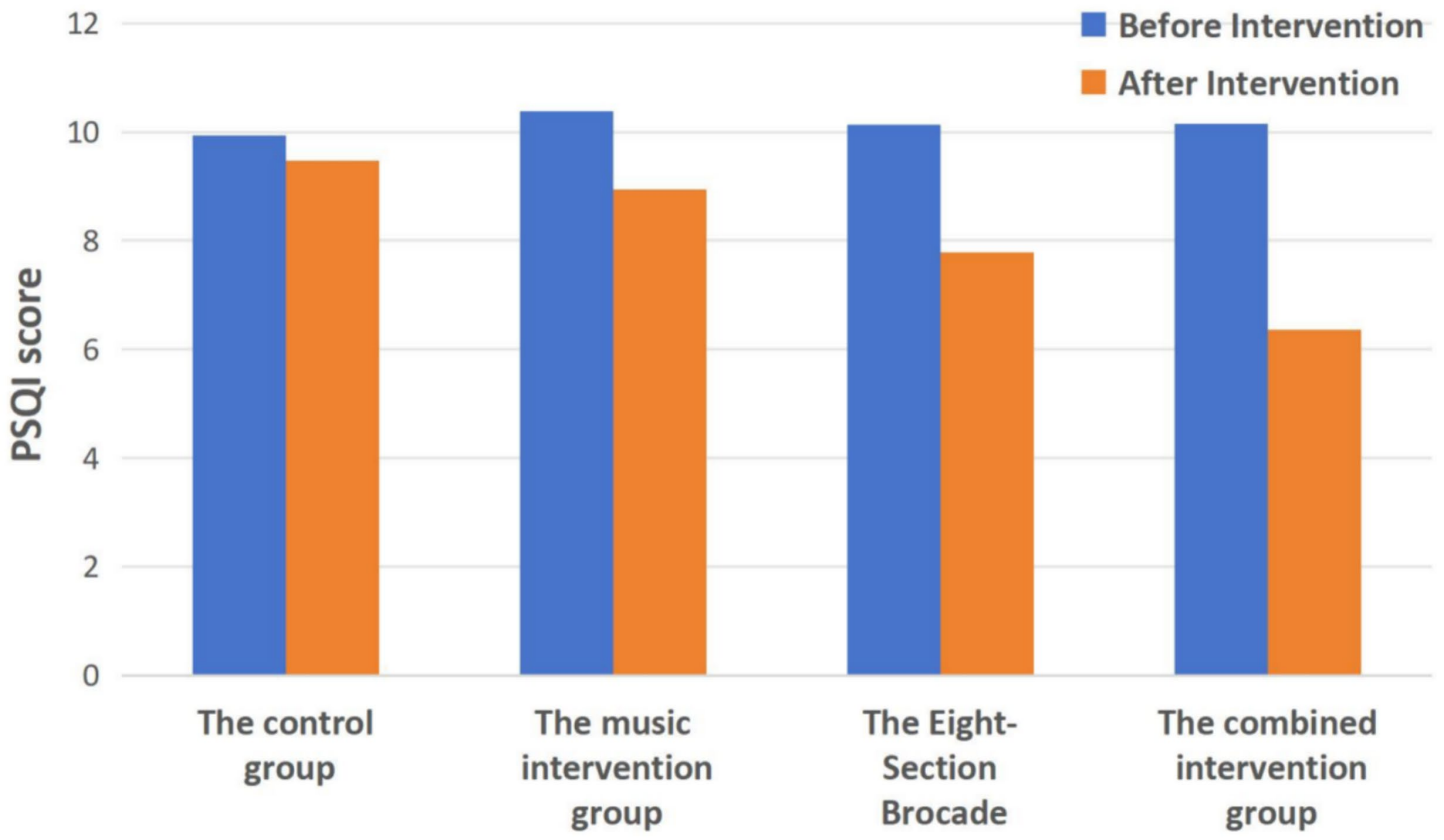
Comparison of PSQI scores before and after intervention in the four groups.

Table 3 and Figure 3 show the comparison of PSQI scores before and after intervention. Before intervention, there was no statistically significant difference in PSQI scores among the four groups (*p* > 0.05). After intervention, compared to before intervention, the PSQI scores of the Music group, Eight-Section Brocade group, and Combined group all significantly decreased (*p* < 0.01). Furthermore, after intervention, the PSQI scores of the Combined group were lower than those of the other three groups. Specifically, the differences between the Combined group and the Control group, as well as between the Combined group and the Music group, were statistically significant (*p* < 0.01), while the difference between the Combined group and the Eight-Section Brocade group was not statistically significant (*p* > 0.05).

**Table 3:** Comparison of PSQI scores before and after intervention in the four groups (Score, $\bar{x}$ ± SD).

Group	Number of cases	Before intervention	After intervention	t/z	p
The control group	36	9.944±2.838	9.472±2.678	1.488	0.146
The music intervention group	37	10.378±3.200	8.944 ± 3.295△△	4.369	0.001
The Eight-Section Brocade	39	10.128±2.922	7.795 ± 2.628△△	−5.060	0
The combined intervention group	39	10.154±2.334	6.359 ± 2.897△△**	9.710	0
*F*/*X*2	–	0.144	23.125	–	–
p	–	0.933	0	–	–

△△ Compared to before intervention within the same group, *p* < 0.01; **Compared to the Control group and Music group after intervention, *p* < 0.01.

## Discussion

Depression is the most common psychological problem among college students. Studies both domestically and abroad have shown that the incidence of depression among college students is higher than that of the general population (Akeman et al., 2020). In China, the prevalence of depression among college students ranges from 13.25 to 79.90% (Zhang et al., 2020). Previous research by the project team indicated that students in medical vocational colleges have a higher incidence of depression compared to undergraduate and graduate students, likely due to their lower educational level, heavy academic workload, shorter study time, and greater employment pressure (Xiong et al., 2019). If left untreated, this could lead to serious consequences.

“Xing Shen He Yi” (Integration of Form and Spirit) is the theoretical basis for TCM in preventing and intervening in depressive emotions. TCM views depressive emotions as a state of functional disorder affecting both the spirit and the form. The principle of nourishing both the form and the spirit is important for preventing and intervening in depressive emotions, addressing depressive emotions from both the physical and mental aspects. Traditional Chinese health-preserving exercises such as the Eight-Section Brocade exercise the body, while traditional Chinese Five-Element Music regulates the spirit, combining movement and stillness to nourish both the form and the spirit.

Five-Element Music Therapy is an ancient and time-honored method of emotional regulation in China, deeply rooted in the theory of the Five Elements of Traditional Chinese Medicine (TCM), particularly the generative and restrictive relationships among the elements. This therapy ingeniously correlates the five musical tones (Gong, Shang, Jue, Zhi, and Yu) with the Five Elements (Earth, Metal, Wood, Fire, and Water). As early as over 2,000 years ago, the medical classic “Huangdi Neijing” (Yellow Emperor’s Inner Canon) clearly expounded the unique theory of “Five Tones Healing Diseases,” which advocates the use of different musical rhythms to balance the functions of the five internal organs, harmonize emotions, and treat illnesses. This therapy not only embodies the holistic perspective and syndrome differentiation treatment principles of TCM but also showcases the extraordinary power of music in promoting physical and mental well-being (Lin and Wu, 2018). According to “SuWen: Chapter Ten, Formation of the Five Zang Organs” it states, “The appearance of the five viscera can be inferred, the sound of the five viscera can be understood, and the color and pulse can be observed. By combining the color and pulse, everything can be thoroughly understood.” In “Ling Shu: The Evil Guests,” the five tones are paired with the five viscera: “The spleen corresponds to Gong, its sound is slow and gentle; the lungs correspond to Shang, its sound is quick and clear; the liver corresponds to Jiao, its sound is forceful and long; the heart corresponds to Zhi, its sound is robust and bright; the kidneys correspond to Yu, its sound is deep and fine.” Based on the characteristics of the five viscera, it is inferred that the five tones correspond to the physiological and pathological conditions of the spleen, lungs, liver, heart, and kidneys. Therefore, the Five-Element Music therapy in TCM acts on the “five viscera” through the five tones, regulating the “five emotions” to achieve the purpose of treatment (Zhang and Liao, 2020; Yuan et al., 2021).

Based on the Five-Element correspondence of the five musical tones, music therapy can regulate the interaction and normal physiological functions of the internal organs, assisting the body in achieving a harmonious state of Yin-Yang balance. TCM’s Five-Element Music exerts its influence on the human body through specific frequency sound waves, adjusting the balance between mind and body, alleviating stress and tension, enabling the body to relax and rest, and ultimately facilitating better adaptation to the environment and daily life (He and Shi, 2024). In clinical practice, the Five-Element Music can not only improve the depressive symptoms and quality of life of elderly patients with depression (Yang et al., 2019), but also alleviate depressive symptoms, improve psychological status, and enhance sleep quality when combined with other methods (Huang et al., 2024; Cao et al., 2024). In addition, the Five-Element Music can also regulate depression and anxiety caused by various diseases, boost patients’ confidence, and improve disease prognosis (Ma et al., 2021; Wu and Yang, 2021; Zhong et al., 2024).

The Eight-Section Brocade, as a health-preserving exercise, emphasizes the integration of “adjusting the body,” “adjusting the breath,” and “adjusting the mind” to strengthen the body and prolong life. The Eight-Section Brocade, rooted in traditional Chinese medical theories, stimulates and adjusts the 12 meridians, Conception and Governor Vessels, acupuncture points, muscles, and bones throughout the body. It gently and smoothly regulates the internal organs, meridians, muscles, and bones, and has the effect of regulating the mind through physical movements and nourishing the spirit through physical activities (Cao et al., 2024). In clinical practice, the Eight-Section Brocade is often used to regulate anxiety and depression associated with various diseases and can improve anxiety and depression in type 2 diabetes patients, aiding in blood sugar control and enhancing patient quality of life (Zu et al., 2021). Zhang et al. (2021) and her team did a meta-analysis on how traditional Chinese exercises like Tai Chi, Eight-Section Brocade, Yijinjing, and Five Animals Frolics can help with anxiety and depression in college students. They found that these exercises can significantly reduce depression and anxiety symptoms, with Eight-Section Brocade and Yijinjing potentially being the most effective.

From the perspective of TCM, Five-Element Music therapy nourishes the spirit through sound, while the Eight-Section Brocade exercises the body through gentle and continuous movements. The combination of Five-Element Music therapy and the Eight-Section Brocade exercise can achieve the goal of “nourishing both the form and the spirit.” In clinical settings, combining Eight-Section Brocade and Five-Element Music therapy can effectively reduce depression in patients with various illnesses and enhance their quality of life (Luo et al., 2021). This combined approach shows better overall improvement than using either method alone (Geng, 2014).

The results of this study show that the SDS scores of the Five-Element Music intervention group, the Eight-Section Brocade intervention group, and the comprehensive intervention group combining Five-Element Music and Eight-Section Brocade all decreased after intervention compared to before intervention within each group (all *p* < 0.01). After intervention, the SDS scores of the music intervention group, the Eight-Section Brocade intervention group, and the comprehensive intervention group were all lower than those of the control group (all *p* < 0.01), and the SDS scores of the comprehensive intervention group were lower than those of the music intervention group and the Eight-Section Brocade intervention group (both *p* < 0.05). This indicates that both the individual Five-Element Music intervention and Eight-Section Brocade intervention, as well as the comprehensive intervention combining Five-Element Music and Eight-Section Brocade, can improve depressive emotions in college students, with the comprehensive intervention being superior to the individual interventions.

Regarding the SAS scores, after intervention, there was a decrease in the Five-Element Music intervention group, the Eight-Section Brocade intervention group, and the comprehensive intervention group compared to before intervention within each group (all *p* < 0.01). After intervention, the SAS scores of the comprehensive intervention group were significantly lower than those of the control group, with a statistically significant difference (*p* < 0.01). Although the SAS scores of the Five-Element Music intervention group and the Eight-Section Brocade intervention group were lower than those of the control group after intervention, the differences were not statistically significant (*p* > 0.05). This suggests that the combined approach of Five-Element Music and Eight-Section Brocade can significantly reduce anxiety in college students, outperforming either intervention alone.

As for the PSQI scores, after intervention, there was a decrease in the Five-Element Music intervention group, the Eight-Section Brocade intervention group, and the comprehensive intervention group compared to before intervention within each group (all *p* < 0.01). After intervention, the PSQI scores of the comprehensive intervention group were significantly lower than those of the control group and the music intervention group, with statistically significant differences (all *p* < 0.01), but there was no statistically significant difference compared to the Eight-Section Brocade intervention group (*p* > 0.05). This suggests that combining Five-Element Music and Eight-Section Brocade effectively enhances sleep quality in college students, while the sole use of Five-Element Music is less effective compared to the comprehensive approach.

Therefore, it can be concluded that the Five-Element Music intervention combined with Eight-Section Brocade, guided by the concept of “Xing Shen He Yi,” is an effective intervention for college students’ depressive emotions, incorporating both dynamic and static components and promoting the integration of form and spirit. The combined effect of the two methods is superior to that of a single method. This approach provides a non-pharmacological intervention strategy and method for preventing and treating depression-related psychological problems in college students, and also offers multiple avenues and methods for regulating and intervening in college students’ psychological problems, thereby safeguarding their psychological well-being. Listening to Five-Element Music is straightforward and practical. Meanwhile, Eight-Section Brocade, a type of fitness qigong, is vigorously advocated by the General Administration of Sport of China. It’s widely acknowledged among college students, often being incorporated into university physical education curricula. Consequently, the comprehensive approach that combines Five-Element Music and Eight-Section Brocade deserves enthusiastic promotion and implementation in universities.

There are still some deficiencies in this study. Firstly, the research subjects only involve one higher vocational college, and there is a certain bias in the selection of research population. In the future, we will strive to promote it to more similar schools and conduct research with larger sample size. Secondly, since the control group did not receive any intervention, there is a concern that the results of the intervention group may be attributed to the “placebo effect” or external factors. In the future, we will optimize the experimental design and design corresponding positive control groups, such as listening to neutral music or engaging in mild physical exercises, to enhance the scientificity of the research. Thirdly, some confounding variables were ignored in the experimental process, such as whether the occurrence of students’ depressive emotions is related to family changes, interpersonal tension, study pressure, employment pressure, etc. These confounding variables should be fully considered in future studies.

## Data Availability

The original contributions presented in the study are included in the article/supplementary material, further inquiries can be directed to the corresponding author.
